# Vacuolar H^+^-Pyrophosphatase AVP1 is Involved in Amine Fungicide Tolerance in *Arabidopsis thaliana* and Provides Tridemorph Resistance in Yeast

**DOI:** 10.3389/fpls.2016.00085

**Published:** 2016-02-09

**Authors:** Agustín Hernández, Rosana Herrera-Palau, Juan M. Madroñal, Tomás Albi, Guillermo López-Lluch, José R. Perez-Castiñeira, Plácido Navas, Federico Valverde, Aurelio Serrano

**Affiliations:** ^1^Instituto de Bioquímica Vegetal y Fotosíntesis, Consejo Superior de Investigaciones Científicas, Universidad de SevillaSevilla, Spain; ^2^Departamento de Parasitologia, Instituto de Ciências Biomédicas, Universidade de São PauloSão Paulo, Brazil; ^3^Centro Andaluz de Biología del Desarrollo and Centre of Biomedical Research in Rare Diseases, ISCIII, Consejo Superior de Investigaciones Científicas, Universidad Pablo de OlavideSevilla, Spain

**Keywords:** pyrophosphate, cell death, abnormal sterols, vacuole, H^+^-pyrophosphatase, V-ATPase, fungicide, crop protection

## Abstract

Amine fungicides are widely used as crop protectants. Their success is believed to be related to their ability to inhibit postlanosterol sterol biosynthesis in fungi, in particular sterol-Δ^8^,Δ^7^-isomerases and sterol-Δ^14^-reductases, with a concomitant accumulation of toxic abnormal sterols. However, their actual cellular effects and mechanisms of death induction are still poorly understood. Paradoxically, plants exhibit a natural resistance to amine fungicides although they have similar enzymes in postcicloartenol sterol biosynthesis that are also susceptible to fungicide inhibition. A major difference in vacuolar ion homeostasis between plants and fungi is the presence of a dual set of primary proton pumps in the former (V-ATPase and H^+^-pyrophosphatase), but only the V-ATPase in the latter. Abnormal sterols affect the proton-pumping capacity of V-ATPases in fungi and this has been proposed as a major determinant in fungicide action. Using *Saccharomyces cerevisiae* as a model fungus, we provide evidence that amine fungicide treatment induced cell death by apoptosis. Cell death was concomitant with impaired H^+^-pumping capacity in vacuole vesicles and dependent on vacuolar proteases. Also, the heterologous expression of the *Arabidopsis thaliana* main H^+^-pyrophosphatase (AVP1) at the fungal vacuolar membrane reduced apoptosis levels in yeast and increased resistance to amine fungicides. Consistently, *A. thaliana avp1* mutant seedlings showed increased susceptibility to this amine fungicide, particularly at the level of root development. This is in agreement with *AVP1* being nearly the sole H^+^-pyrophosphatase gene expressed at the root elongation zones. All in all, the present data suggest that H^+^-pyrophosphatases are major determinants of plant tolerance to amine fungicides.

## Introduction

Ion-translocating pyrophosphatases are a type of primary transporters that exemplifies a key difference between plants and fungi. In the latter, similar to what is found in animals, PPi is hydrolysed to orthophosphate solely by means of soluble pyrophosphatases and the energy in the phosphoanhydride bond is released as heat. On the other hand, in plants and other organisms, ion-translocating pyrophosphatases couple the hydrolysis of PPi to the transport of ions across membranes. Additionally, other enzymes use pyrophosphate. PPi-dependent phosphofructokinase (PFP, or diphosphate:D-fructose-6-phosphate 1-phosphotransferase) and UDP-glucose pyrophosphorylase have a direct role in PPi and sucrose homeostasis (van der Merwe et al., 2010; Wang et al., 2013). This has obvious benefits for the plant cell in terms of energetics but also in terms of ion homeostasis. In all eukaryotes, V-ATPases are a different type of H^+^ pumps that drive the generation of a H^+^-gradient utilizing ATP as a substrate. Notoriously, these pumps are found in plant cells in the same organelles where H^+^-PPases are present, in brief, the vacuole, Golgi apparatus, and endosomes (Segami et al., 2010, 2014). To date, the implications of the presence of a double set of pumps in plant cells is still an unresolved issue, although a role in stress resistance has been proposed (Atwell et al., 2015).

Fungal pests are the greatest biological challenge in crop protection since they comprise the largest number of plant pathogens. Treatment of fungal plant diseases often relies on fungicides as one of its most effective defenses and, in this respect, ergosterol biosynthesis inhibitors (EBIs) are among the most important of fungicides in terms of market share. Two main kinds of EBI are usually considered: azoles, which target the product of the *ERG11* gene, and amines (formerly morpholines) that exert their inhibition on the products of the genes *ERG24* and *ERG2*. It has been shown that fungicides inducing the accumulation of Δ^14^-sterols and 14α-methylated sterols, through Erg24p and Erg11p inhibition respectively, exert part of their effects *via* inhibition of the vacuolar H^+^-ATPase in *Saccharomyces cerevisiae* and *Candida albicans* (Zhang and Rao, 2010; Zhang et al., 2010). Similarly, yeast mutants bearing mutations in the *ERG2* gene has been shown to display faulty V-ATPase-dependent proton pumping and intraorganellar acidification leading to a plethora of cellular defects (Hernandez et al., 2015). The *S. cerevisiae ERG2* gene encodes the yeast sterol-Δ^8^,Δ^7^-isomerase, an ortholog of *Arabidopsis thaliana HYD1* (Souter et al., 2002) and it is a target for amine fungicides like tridemorph. Noticeably, this fungicide is specific for Erg2p in yeast (Baloch and Mercer, 1987), while fenpropimorph and other amine fungicides also target Erg24p. Effects of a gene mutation and its corresponding fungicide do not always concur. Thus, *Ustilago maydis* strain P51, an *erg2^-^* mutant, showed a reduced change in fatty acid unsaturation and a lesser activation of the plasma membrane H^+^-ATPase than an amine fungicide-treated wild type as a result of the accumulation of 8-dehydrosterols (Hernandez et al., 1997). It is therefore necessary to evaluate the effects of fungicides in appropriate systems. To date, the mechanism of proliferation control by amine fungicides is still unclear. In addition, the prevalent hypothesis on the mechanism of fungal cell death induction by EBIs posits that abnormal sterols induce permeabilisation and dysfunction of the plasma membrane (Van den Bossche et al., 1983). However, this hypothesis was formulated before the introduction of modern concepts in programmed cell death processes (PCDs) like apoptosis, necrosis or mitotic catastrophe and, since then, PCD has been described in some instances of EBI use on parasitic fungi (Ramsdale, 2008). Paradoxically, plants and fungi have been known for a long time to display similar *in vitro* inhibitor sensitivity in their sterol biosynthetic enzymes (Rahier et al., 1986), but the latter organisms are far more sensitive to inhibition of this pathway and no cell death-associated mechanism has been described in plants. On the other hand, EBIs have been described to exert a toll on plant growth and development (Buchenauer and Röhner, 1981; Köller, 1987; Cerdon et al., 1996).

In the present report, we provide evidence showing the stimulation of apoptotic cell death by the amine fungicide tridemorph in yeast and that its mechanism is, in part, related to inhibition of vacuole acidification. Also, we present data showing that H^+^-PPases in plants may play as fail-safe devices in tridemorph tolerance and help counteracting fungicide harmful effects such as inhibition of root development. We also provide evidence on the distribution of the expression of the three different H^+^-PPase gene isoforms in the model plant *A. thaliana* which could help to understand plant tolerance to amine fungicides.

## Materials and Methods

### Yeast Strains, Plasmids, and Growth Conditions

All strains are derivatives of *S. cerevisiae* strain W303-1a (MATa *leu2-3,112 trp1-1 can1-100 ura3-1 ade2-1 his3-11,15*). Relevant genotypes are described in **Table 1**. Yeast soluble PPase *IPP1* or TcGFP-AVP1, a chimera consisting of the main H^+^-PPase vacuolar isoform AVP1 from *A. thaliana* fused to the *N*-terminal sequence of the H^+^-PPase TcVP from the protist *Tripanosoma cruzi* and the sequence of the GFP from *Aequorea victoria*, were expressed from a pRS699 multicopy plasmid as described (Drake et al., 2010). Introduction of plasmids into yeast cells was done by the lithium-acetate method (Gietz and Woods, 2002). Cells were routinely grown on standard YP or synthetic media supplemented with appropriate carbon sources (Sherman, 1991). Unless otherwise stated, all determinations were done on exponentially growing cells (0.5 < A_600_ < 0.8). For drop tests, cells were grown to early stationary phase; A_600_ of cultures was adjusted to 0.4 (*ca* 4 × 10^6^ cells/ml) with water and three 10-fold serial dilutions in water prepared from them. Two-point-five microliter aliquots from each dilution were placed onto appropriate agar plates, resulting in *ca* 10^4^, 10^3^, 10^2^, or 10 cells per spot, accordingly. Liquid cultures were routinely treated with 3 μM tridemorph for 3 h (annexin V experiments) or 5 h (cell cycle analysis) prior analysis.

**Table 1:** Yeast strains.

Name	Relevant genotype	Source
W303-1	MATa *leu2-3,112 trp1-1 can1-100 ura3-1 ade2-1 his3-11,15*	Davies et al., 2005
YPC3	W303-1*a ipp1_UAS_-ipp1_TATA_::HIS3-GAL1_UAS_-GAL1_TATA_-IPP1*	Drake et al., 2010
SAH6	YPC3 *vph1Δ*::KanMX4	Hernandez et al., 2015
SAH11	W303-1a *yca1Δ*::KanMX4	This work
SAH13	W303-1a *pep4Δ*::*TRP1*	This work

### Vacuole Vesicles and ATPase Assays

Intact vacuoles were isolated essentially as described (Hernandez et al., 2009) with the only modification of using a single 8% (w/w) Ficoll gradient. After isolation, vacuoles were vesiculated by resuspension in TKE buffer [10 mM Tris/HCl pH 7.5, 2 mM KCl, 1 mM EDTA and protease inhibitor cocktail (Sigma-Aldrich)]; vesicles were recovered after centrifugation at 100,000 × *g* for 30 min and resuspended in TKE. The formation of a ΔpH was evaluated as the ACMA fluorescence quenching produced by the activity of the tonoplast H^+^-ATPase using the following reaction mixture: 1 μM ACMA, 20 mM MOPS-Tris pH 7.2, 25 mM KCl, 2 mM MgCl_2_, and vacuolar membrane preparation (up to 50 μg of protein). The assay was initiated with the addition of 1.5 mM ATP. Recovery of fluorescence, as a proof of gradient formation, was induced by adding 3 μM gramicidin D. ATPase hydrolytic activities were assayed as described (Hernandez et al., 1994).

### Flow Cytometry

Annexin-V-FITC and PI staining test were done essentially as described in Madeo et al. (1997). Briefly, after treatment, cells were collected, washed once in distilled water, once in SPMB (1.2 M sorbitol, 50 mM phosphate buffer pH 7.5, 1 mM MgCl_2_) and resuspended in SPMB with 30 units of lyticase (Sigma, St. Louis, MO, USA) per 10^7^ cells. After protoplasting was complete (0.5–1 h), protoplasts were collected by centrifugation for 5 min at 500 × *g* and washed once with fresh SPMB and a second time with annexin-V labeling buffer (1.2 M sorbitol, 10 mM HEPES-NaOH pH 7.4, 140 mM NaCl, 2.5 mM CaCl_2_). Annexin-V-FITC and PI (final concentrations 1 μg/ml and 2.5 μg/ml, respectively) were incubated with protoplasts in labeling buffer for 20 min prior to flow cytometry. For DNA content analysis, cells were stained with PI essentially as described by Sazer and Sherwood (1990), and analyzed on a Coulter Epics XL apparatus as previously described (Hernandez et al., 2008). Although the extent of death induction by tridemorph was somewhat batch-dependent, the phenomena were consistent and reproducible within each batch.

### Plant Materials and Growth Conditions

Plant mutant genotypes are described in **Table 2**. All *A. thaliana* wild-type and mutants were from Columbia ecotype. Seeds were sterilized using vapor phase-chlorine gas for 5 h (Clough and Bent, 1998) and sown individually using sterile forceps on 1X Murashige and Skoog (MS) plates containing 1% sucrose and 3.5 g l^-1^ Phytagel (Sigma-Aldrich).

**Table 2:** Plant mutants.

Mutant	Gene	Protein	Mutation	Reference
*fugu 5-1*	At1g15690	AVP1	A709-T	Ferjani et al., 2011
*fugu 5-3*	At1g15690	AVP1	A553-T,Δ554-558	Ferjani et al., 2011
*avp2-1* (*sm1*)	At1g78920	AVP2	Fifth intron	This work
*avp2-2* (*sm2*)	At1g78920	AVP2	3′ UTR	This work

Homozygous mutant lines for gene At1g15690 encoding the vacuole-localized H^+^-PPase *AVP1* isoform (*fugu 5-1* and *fugu 5-3*) were a kind gift of Dr Ferjani (Tokyo Gakugei University, Tokyo, Japan). Mutants for gene At1g78920 encoding the *AVP2* isoform were obtained from the collection held at the Salk Institute Genomic Analysis Laboratory (USA) and named for this work as *sm1* and *sm2*. Homozygosis of the T-DNA insertion was confirmed by PCR. Details on mutants are listed on **Table 2**.

### Histological Determination of Gene Expression

Promotor sequences from genes *AVP1* (At1g15690, 1,930 bp upstream of ATG), *AVP2* (At1g78920, 1,100 bp), and *AVP3* (At1g16780, 900 bp) were amplified by PCR from genomic DNA and cloned into vector pMDC162 (Curtis and Grossniklaus, 2003). Columbia wild-type *A. thaliana* plants were transformed by the floral dipping method (Clough and Bent, 1998). Plants carrying gene insertions were selected on kanamycin-supplemented plates. All experiments were performed with F3 seeds. The transgene insertions in all lines were sequenced to check for integrity of the inserted constructs. Staining was done by immersing plants or seedlings in GUS buffer [2 mM 5-bromo-4-chloro-3-indol-β-D-glucuronic acid, 50 mM potassium phosphate buffer (pH 7.2), 2 mM potassium ferrocyanide, 2 mM potassium ferrycyanide, 0.2% (w/v) triton X-100] for 1–3 days. Chlorophyll was washed-off using several 96% (v/v) ethanol washes at 37°C. Images were collected using a Nikon Eclipse 80i microscope coupled to a Nikon Digital Sight DS L1 camera or with a Leica MZFLIII binocular coupled to a Leica DC camera.

### Semi-quantitative Reverse Transcription-PCR

Total RNA (1 μg) was subjected to cDNA synthesis using a “QuantiTect^®^ Reverse Transcription kit” (Qiagen) according to manufacturer’s instructions. Final cDNA (1 μl) was subjected to PCR using primers amplifying 300–400 bp fragments at the 3′ UTR regions of the cDNA corresponding to genes At1g15690 (*AVP1*), At1g78920 (*AVP2*), At1g16780 (*AVP3*), and At4g05320 (*UBIQUITIN10, UBQ10*). PCR was run for a total of 20 cycles. Primers annealing on the *UBQ10* cDNA were used to normalize equal cDNA loading onto PCR mixes. Saturation of the reaction and specificity of primers were checked using a five-fold excess of cDNA.

### Plant Extracts

Seedlings were frozen in liquid nitrogen and kept at -80°C until use. Seedlings were ground with pestle and mortar in liquid nitrogen. The powder was resuspended in 1 ml of extraction buffer containing 50 mM MOPS adjusted to pH 7.5 with NaOH, 330 mM sorbitol, 2 mM MgCl_2_, 1 mM DTT, 1 mM PMSF and Plant Protease Inhibitor Cocktail (Sigma-Aldrich). The mixture was centrifuged for 15 min at 16,000 rpm at 4°C, the supernatant transferred to a new tube and reserved for Western blot analysis.

### Hypocotyl and Root Length Measurements

To measure hypocotyl and root elongation in the darkness, seeds were stratified for 5 days at 4°C in the dark before being placed vertically in a growth chamber equipped with fluorescence light (light intensity 100 μE m^-2^ s^-1^) at 22°C for 4 h. To ensure dark conditions after that period, plates were wrapped in four layers of aluminum foil and plates incubated vertically at 22°C for 6 days. When appropriate, plates were supplemented with 10 μM tridemorph. Fungicide treatments and mock controls were carried out simultaneously in three replicate experiments. Hypocotyl and root length were quantified using Image J software (Schneider et al., 2012).

### Protein Determination and Western Blotting

Protein determination was done using a dye-binding based assay from Bio-Rad (Hercules, CA, USA), according to manufacturer instructions or a modified Lowry assay (Thermo Scientific, Rockford, IL, USA), using ovoalbumin as a standard. Proteins were separated in SDS-PAGE gels using standard procedures (Sambrook et al., 1989). Proteins were then transferred to nitrocellulose filters and probed with polyclonal antibodies raised in rabbit against VHA-A (Cosmobio, Tokyo) and AVP1 (Agrisera, Vånnås) at a 1:1000 dilution. Blots were visualized on X-ray films using horseradish peroxidase-coupled secondary antibodies and a chemiluminescence kit (Millipore, Madrid). Quantification of blot band intensity was done using a Bio-Rad GS-800 densitometer.

## Results

### The Amine Fungicide Tridemorph Induces Metacaspase-Independent Apoptosis

**FIGURE 1:**
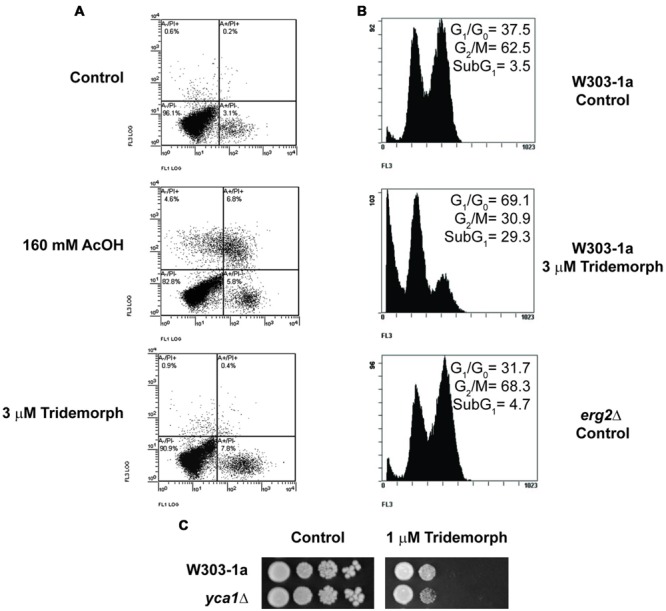
**Cell death and cell cycle arrest induction in tridemorph treated yeast cells. (A)** Determination of early apoptosis and necrosis in tridemorph-treated yeast. Externalization of phosphatydilserine and plasma membrane permeability to propidium iodide (Annexin V-FITC/PI assays) assessed by flow cytometry in non-permeabilised yeast protoplasts obtained from cells treated with 3 μM tridemorph for 3 h. Acetic acid treatment is included as a control inducing both Annexin V-FITC and PI fluorescence. **(B)** Cell cycle profiles in tridemorph treated yeast cells; quantitation of cell death. Flow cytometry determination of DNA contents by propidium iodide staining in ethanol-permeabilised yeast cells. Percentages of cells in every stage are shown in each panel. Total cells analyzed per case: 10,000. **(C)** Tridemorph-induced apoptosis dependence from metacaspases. Early stationary yeast cultures were diluted and spotted onto YPD agar plates (control, left) and YPD plus 1 μm tridemorph (right) at densities distributing *ca* 10^4^, 10^3^, 10^2^, and 10 cells per spot, from left to right, respectively.

We aimed at determining if inhibition of *ERG2* gene product was fungistatic or if it induced cell death, and in the latter case, which type of cell death. Therefore, we treated W303-1a cells with 3 μM tridemorph for 5 h prior to annexin V-FITC/PI flow cytometric tests (**Figure 1A**). Both annexin-V-FITC (AV) and PI are membrane impermeable molecules; AV is a protein that binds phosphatidyl serine, while PI dyes nucleic acids. Briefly, cells dying from apoptosis expose phosphatidyl serine on the outer leaflet of the plasma membrane early in the process and only become permeable to PI at late stages, while necrotic cells are characterized by early breakage of the plasma membrane permeability barrier and little or no translocation of phosphatidyl serine (Madeo et al., 1997). Yeast cells treated with a growth inhibitory dose of tridemorph for 3 h displayed a differential staining with AV but little staining with PI in non-permeabilized protoplasts, compared with an untreated control (**Figure 1A** and **Table 3**). In comparison, cells treated with 160 mM acetic acid, a condition known to induce both apoptosis and necrosis in yeast (Ludovico et al., 2001) yielded a significant fraction of the protoplasts permeable to PI (**Figure 1A**). Accumulation of abnormal sterols has been associated to cell cycle perturbations (Marcireau et al., 1990; Fernández et al., 2002); hence, we also investigated if cell cycle arrest occurred concomitantly to apoptosis. Experiments using PI staining of permeabilised cells previously treated with tridemorph revealed that genomic DNA fragmentation takes place upon fungicide treatment with an increase in cells at the G1 phase of the cell cycle (**Figure 1B**). On their turn, *erg2Δ* (JRY7773) cells showed no differences with respect to a wild-type strain in subG_1_ extent (below 4%) and only a slight accumulation of cells in G_2_/M phases of the cell cycle, compared with W303-1a. Deletion of YCA1, the only yeast metacaspase, on a W303-1a background yielded cells with no differential sensitivity toward tridemorph as assessed by drop test assays (**Figure 1C**) or PI-flow cytometry of permeabilised cells (data not shown).

**Table 3:** Annexin V-FITC and propidium iodide staining of W303-1a yeast cells.

	AV/PI staining			
	-/-	+/-	-/+	+/+
Untreated	94.2 ± 3.1	3.0 ± 0.3	0.7 ± 0.0	0.1 ± 0.0
3 μM Tridemorph	91.3 ± 0.0	7.4 ± 0.1	0.9 ± 0.1	0.4 ± 0.0

*Cells were treated with with tridemorph for 5 h prior to analysis.*

*AV, annexin V-FITC; PI, propidium iodide. Data in percentage ± SEM.*

### Tridemorph Causes V-ATPase Dysfunction

**FIGURE 2:**
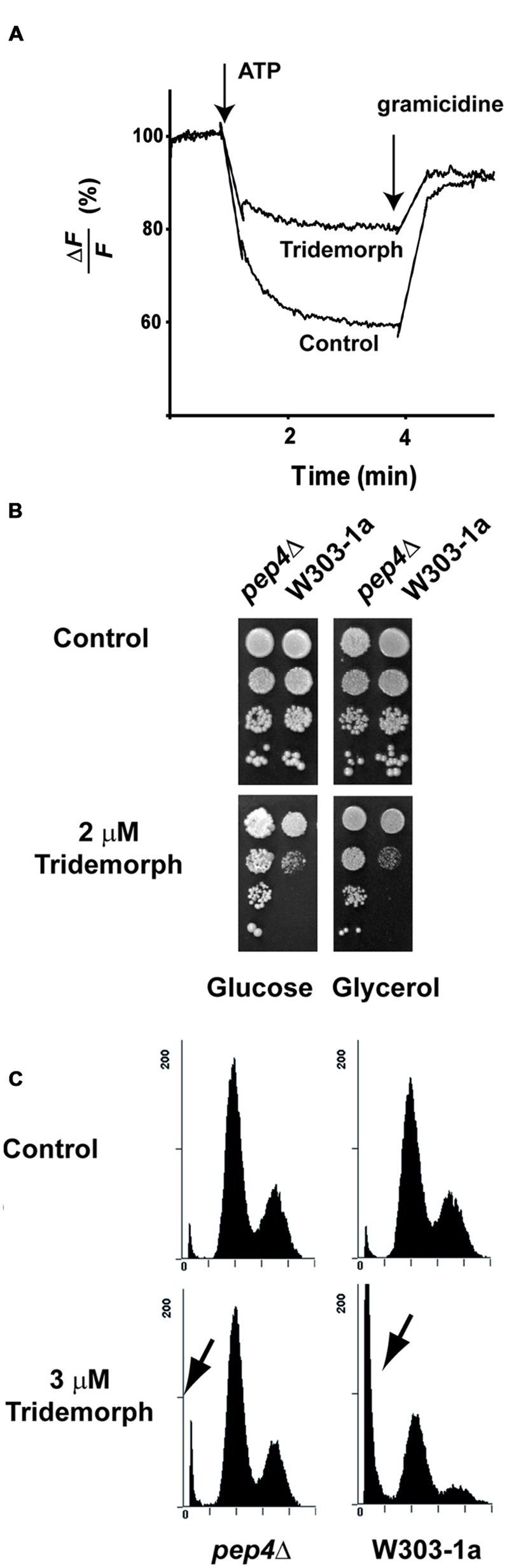
**Vacuolar V-ATPase inhibition by tridemorph treatment. (A)** Proton pumping assays using vacuolar vesicles. Proton transport in vacuole vesicles isolated from control yeasts (lower trace) and treated with 3 μM tridemorph for 5 h (top trace) followed by quenching of ACMA fluorescence in the presence of Mg⋅ATP **(B)** Enhancement of tridemorph tolerance in yeast by deletion of the gene corresponding to Proteinase A (*PEP4*). Early stationary yeast cultures were diluted and spotted onto control (upper panels) or 2 μm tridemorph-supplemented (lower panels) YPD (Glucose) or YPGlycerol agar plates at densities distributing *ca* 10^4^, 10^3^, 10^2^, and 10 cells per spot, up to down, respectively. **(C)** Quantitation of cell death in *pep4Δ* mutant cells by flow cytometry. Flow cytometry determination of DNA contents by propidium iodide staining in ethanol-permeabilised *pep4Δ* mutant (left) and W303-1a (right) yeast cells in control (upper panels) and 3 μm tridemorph-treated cultures (lower panels). Arrows point to dead cell populations (Sub-G_1_). A total of 10,000 cells were analyzed per case per experiment.

Tridemorph is a fungicide that phenocopies a defect on *ERG2*, which causes V-ATPase dysfunction (Hernandez et al., 2015). Bearing in mind the differences found in terms of cell death induction between tridemorph treatment and an *erg2Δ* mutation, we were interested in ascertaining if inhibition of V-ATPase proton pumping was reproduced by tridemorph (**Figure 2**). Vacuole vesicles extracted from cells treated with 3 μM tridemorph for 5 h were not capable to form proton gradients (**Figure 2A**). This impairment was similar using *erg2Δ*-derived vesicles (Hernandez et al., 2015). Also in agreement with previous results, hydrolytic activity assays showed that V-ATPase activity was inhibited to a minor extent (435 ± 97 vs. 341 ± 8 μmol Pi min^-1^ mg^-1^ protein ± SEM for vesicles obtained from control and tridemorph-treated yeast cells respectively).

In a previous report (Hernandez et al., 2015), we described that the presence of 8-dehydrosterols in yeast membranes induced a change in subunit a of the V-ATPase that made it more susceptible to proteolytic attack. We hypothesized that limiting the proteolytic turnover of vacuolar proteins might help yeast to withstand tridemorph treatment. To evaluate this hypothesis, we deleted *PEP4*, the gene encoding vacuolar protease A, since this deletion is known to affect a wide range of proteolytic activities in this organelle (Ammerer et al., 1986; Woolford et al., 1986). In addition, defects on vacuole acidification are associated to deficiencies to grow on non-fermentable carbon sources (Kane, 2006). Spot tests using W303-1a and its *pep4Δ* derivative showed that deletion of this protease provided a proliferative advantage when cells were challenged with tridemorph on agar plates, both on glucose and on glycerol carbon sources (**Figure 2B**). We also tested if cell death induction or cell cycle were affected in these cells. Analysis of PI-stained permeabilised yeast cells by flow cytometry showed again a modest accumulation of cells in G_1_/G_0_ phases, irrespectively of the genotype (**Figure 2C**, upper panels). However, while proportions of cells in subG_1_ raised from 4.5 ± 0.4% to 47.6 ± 0.5% in the case of W303-1a cells treated with vehicle or tridemorph, respectively, *pep4Δ* cells showed only 4.9 ± 0.3% and 7.3 ± 0.4% for vehicle and fungicide-treatments, respectively (**Figure 2C**, lower panels).

### A Heterologously Expressed Plant H^+^-PPase Gene Alleviates Tridemorph Growth Inhibition and Cell Death in Yeast

**FIGURE 3:**
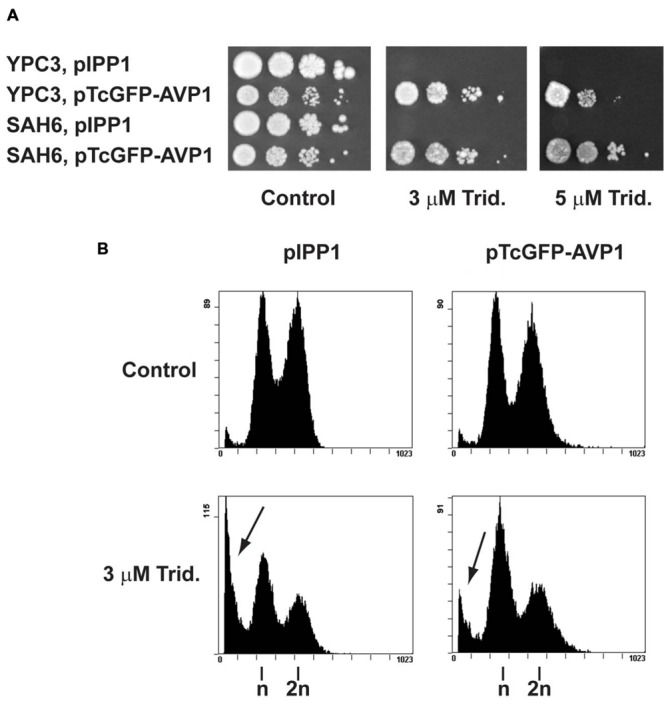
**Influence of the heterologous expression of a H^+^-PPase gene on tridemorph tolerance in yeast cells. (A)** Evaluation of tolerance to tridemorph in wild-type type (YPC3) and V-ATPase (SAH6) mutants. Early stationary yeast cultures were diluted and spotted onto control (left), 3 μm tridemorph-supplemented (middle) and 5 μm tridemorph-supplemented (right) YPD plates at densities distributing *ca* 10^4^, 10^3^, 10^2^, and 10 cells per spot, respectively. **(B)** Quantitation of cell death in yeasts expressing a functional plant H^+^-PPase gene by flow cytometry. Flow cytometry determination of DNA contents by propidium iodide staining in ethanol-permeabilised yeast cells expressing episomally *IPP1* (left panels) and plant *AVP1* (right panels). Arrows point to dead cell populations (Sub-G_1_). A total of 10,000 cells were analyzed per case per experiment.

Since tridemorph induced cell death in yeast cells but deletion of *ERG2* did not reproduce this effect, we were interested to know if an abnormal vacuolar acidification could be involved in the stimulation of cell death by tridemorph. To this end, YPC3 cells were transformed with pTcGFP-AVP1 or pIPP1 plasmids and kept on glucose medium to repress the expression of the genomic *IPP1* gene encoding a soluble pyrophosphatase (**Figure 3**). Overexpression of the *A. thaliana* H^+^-PPase chimera *TcGFP-AVP1* conferred resistance to concentrations of this amine fungicide above those able to restrict growth to wild-type cells (**Figure 3A**). This resistance could also be observed and was slightly greater in strains lacking a vacuole-localized V-ATPase (**Figure 3A**, rightmost panel). Under the present conditions, tridemorph treatment did not affect substantially the H^+^-pumping ability of TcGFP-AVP1 (Supplementary Figure S1).

In order to obtain a more direct and quantifiable measurement of apoptosis, YPC3 cells transformed with pTcGFP-AVP1 or pIPP1 plasmids were treated with tridemorph in liquid culture for 5 h. Cell cycle was then analyzed by PI-flow citometry (**Figure 3B**). Tridemorph treatment induced changes in cell cycle profiles leading to an accumulation of cells in the G_1_/G_0_ phase of the cell cycle, irrespectively of the plasmid borne. This was akin to what was observed in W303-1a cells (**Figure 1B**), although reduced in extent. In particular, the proportion of cells in G_1_/G_0_ was 42.8 ± 1.8% and 55.3 ± 0.8% for *IPP1*-expressing cells untreated or treated with 3 μM tridemorph, respectively. Cells expressing *TcGFP-AVP1* showed similar proportions: 45.7 ± 2.2% vs. 52.4 ± 0.1% in G_1_/G_0_ for untreated and tridemorph-treated cells, respectively. As previously shown, tridemorph induced a dramatic increase in cell death as assessed by accumulation of cells with subG_1_ complements of genomic DNA. Thus, pIPP1-transformed YPC3 strain showed 3.7 ± 0.2% of cells in subG_1_ when untreated but this proportion raised up to 29.0 ± 2.8% upon tridemorph treatment. However, presence of an H^+^-PPase as an alternative proton pump in YPC3 alleviated this cell death induction (4.2 ± 0.2% vs. 14.3 ± 2.8% in untreated and tridemorph-treated pTcGFP-AVP1 YPC3 cells, respectively).

### Expression of H^+^-PPases Gene Isoforms in *Arabidopsis thaliana*

*Arabidopsis thaliana* genome includes three different genes encoding membrane-bound H^+^-translocating pyrophosphatases (*AVPs*), with gene products named AVP1 (type I or K^+^-stimulated isoform), and AVP2, AVP3 (type II, K^+^-independent isoforms). Bearing in mind the observations made in yeast, we hypothesized that these proteins could play a major role in fungicide tolerance in this model plant. However, in order to make meaningful functional assignments, a previous comparative study on the expression of these genes was necessary. Although some studies have already been published (Mitsuda et al., 2001a,b; Segami et al., 2010; Pizzio et al., 2015), none of them made a comparative study, nor included *AVP3* expression. We met this task by making transgenic lines with *AVP-promoter*-β-glucuronidase (GUS) reporter system, where the different *AVPs* promoters were genomic fragments encompassing 1930, 1100, and 930 bp upstream the ATG codon of *AVP1, AVP2*, and *AVP3* genes, respectively. Qualitative results are summarized on **Table 4**.

**Table 4:** Location of H^+^-PPase mRNA expression.

	Cot.	Root	Stem	Mer.	R.L.	C.L.	Hyd.	G.C.	Pist.	Sta.	I.L.	Sil.
*AVP1*	-	+	+	+	**-**	+/-	+	+	+	+	+	+/-
*AVP2*	-	+	-	+	-	-	+	-	+	-	+	+/-
*AVP3*	-	+/-	-	+/-	-	-	+/-	+/-	+/-	-	+/-	-

*Cot., Cotyledon; Mer., apical meristem; R.L., Rosette leave; C.L., Caulinar leaf; Hyd., hydathodes; G.C., guard cells; Pist., pistil; Sta., stamen; I.L., inflorescence leaf; Sil., siliqua.*

**FIGURE 4:**
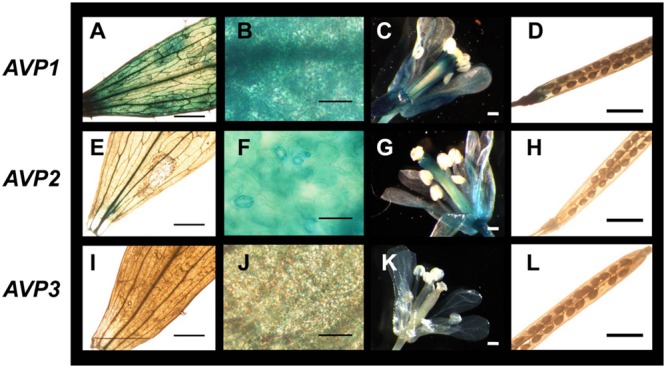
**Expression of H^+^-PPase genes in adult organs and tissues of *Arabidopsis thaliana*. (A,E,I)** Cauline leaves; **(B,F,J)** detail of cauline leaves (mesophyll); **(C,G,K)** inflorescences; **(D,H,L)** silique. Transgenic plants expressing β-glucuronidase as a reporter of the expression driven from a 1.9, 1.1, or 0.9 kb fragment from the *AVP1, AVP2*, or *AVP3* promotor sequence, respectively. Bars correspond to 0.5 mm **(A,C–E,G–I,K,L)** or 50 μm **(B,F,J)**.

In adult plants (**Figure 4**), *AVP1* was expressed mostly at the vascular tissues but at low levels in leaf mesophyll. In inflorescences, its expression was evident in pistils, stamen pedicels and the vascular system of sepals, while its expression in siliqua was only significantly observed at its base (**Figures 4A–D**). GUS staining corresponding to *AVP2* (**Figures 4E–H**) was also present in the vascular system, albeit to a smaller degree than *AVP1* (**Figure 4E**). Leaf expression was again very low in mesophyll and, in addition with vascular bundles, only clearly observed in guard cells (**Figure 4F**). In reproductive tissues, its signal was perceptible at the top and bottom parts of the pistil, in stamen pedicels and vascular tissue of sepals (**Figure 4G**), while in siliqua, the signal was nearly undetectable (**Figure 4H**). GUS signal corresponding to *AVP3* expression was very weak throughout the plant and down to undetectable levels in many tissues. However, some signal was apparent in reproductive tissues, particularly a weak expression was observed in pistils, stamen pedicels and sepals (**Figure 4K**).

**FIGURE 5:**
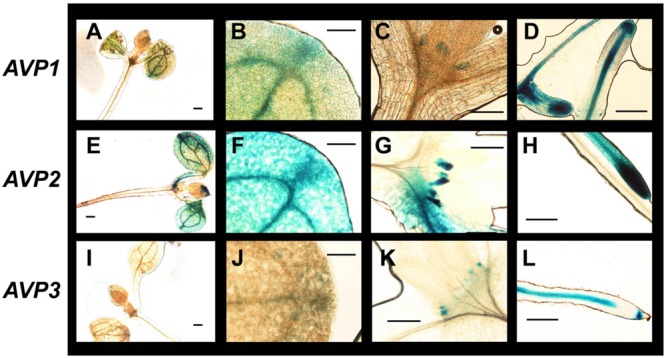
**Expression of H^+^-PPase genes in seedling organs and tissues of *A. thaliana*. (A,E,I)** Whole seedling; **(B,F,J)** detail of rosette leaves showing hydathodes; **(C,G,H)** shoot apical meristems; **(D,H,L)** root. Transgenic plants expressing β-glucuronidase as a reporter of the expression driven from a 1.9, 1.1, or 0.9 kb fragment from *AVP1* (upper panel), *AVP2* (middle panel), or *AVP3* (lower panel) promotor sequences, respectively. Plants were incubated with X-Gluc to develop the signal prior to being photographed. Bars correspond to 0.1 mm **(A,D,E,H,I,L)** or 50 μm **(B,C,F,G,J,K)**.

In general, expression was more evident in developing seedlings for all three isoforms (**Figure 5**). *AVP1* was robustly expressed in roots and stems and, especially, at the root columella and apical meristem (**Figures 5A–D**). In roots, the signal was easily detectable up to the transition zone with the shoot (**Figure 5D**). On the other hand, developing leaves and meristems showed high levels of GUS staining, particularly at the hydathodes (**Figure 5B**). *AVP2* signal (**Figures 5E–H**) was found in leaf and root apices, although at a lower intensity to *AVP1* and often showing intermissions in non-dividing tissue (**Figure 5E**). Also, it was detected in developing apical meristems (**Figure 5G**). Leaf expression was again observed in developing organs, remarkably at the hydathodes and in comparable levels to that of *AVP1* (compare **Figures 5B–F**). In the case of *AVP3* expression (**Figures 5I–L**), a clear signal was only observed in the apical meristem (**Figure 5K**) and in the root vascular system (**Figure 5L**); in leaves, no clear signal was detected (**Figure 5J**).

**FIGURE 6:**
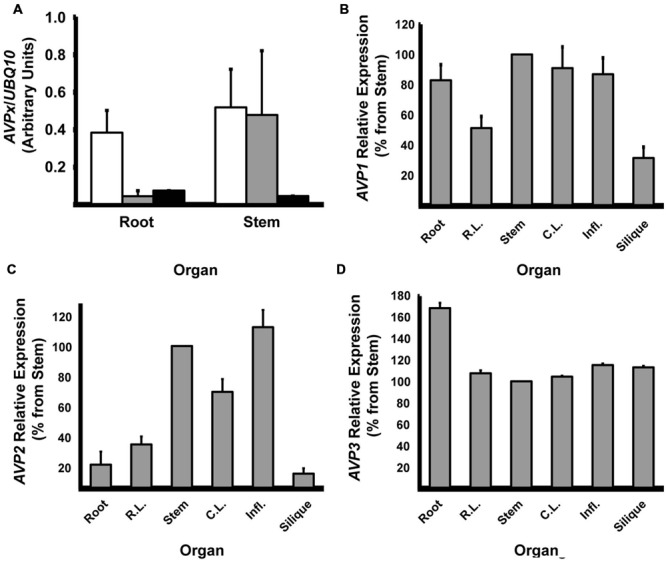
**Relative expression of the different H^+^-PPase isoforms. (A)** Expression of *AVP1-3* in adult root and stem tissues. Open bars: *AVP1*; Shaded bars: *AVP2*; Black bars: *AVP3*. **(B)** Relative expression of *AVP1* in different plant organs. **(C)** Relative expression of *AVP2* in different plant organs. **(D)** Relative expression of *AVP3* in different plant organs. Semi-quantitation of mRNA abundance estimated by RT-PCR. Data were normalized against the expression of *UBQ10* gene. **(B–D)** Average expression in stems was taken as 100%. Expression (*AVPx*/*UBQ10*) in stems were *AVP1*: 0.52 ± 0.21; *AVP2*: 0.48 ± 0.36; *AVP3*: 0.04 ± 0.00. Data are mean ± SE of three independent experiments.

Since GUS staining indicated some clear differences in the expression of the three *AVPs*, and in order to confirm the data, semi-quantitative RT-PCR experiments were used (**Figure 6**). First, we confirmed that there were large differences in the expression of the isoforms. We chose tissues of adult plants grown on soil where the most diverse differences in GUS staining had been observed and, in particular, two tissues where the *AVPs* expression mostly differed: roots and stems (**Table 4**). The expression of the gene *UBQ10* encoding Ubiquitin-10 was used as a loading control. *AVP1* mRNA was more abundant than any of the other two isoforms in roots, but *AVP1* and *AVP2* transcripts showed comparable levels in stems. On the other hand, *AVP3* mRNA was nearly undetectable in both tissues (**Figure 6A**).

We next wanted to confirm that the expression of each *AVP* gene varied widely between organs. On the whole, *AVP1* mRNA relative amount estimates agreed with GUS data. Thus, it showed a high level of expression in roots, stems and influorescences and minimal in siliques and rosette leaves. Curiously, cauline leaves showed a strong *AVP1* expression, in contrast to GUS data. All in all, little differences in expression were found among organs and tissues with values near those found in stems (**Figure 6B**). *AVP2* mRNA showed a relatively robust expression in stems and inflorescences and again in cauline leaves. Minimal levels of expression were observed in roots, rosette leaves and siliques. In this case, the differences between high- and low-expression tissues were greater than in the case of *AVP1* (**Figure 6C**). Regarding *AVP3* expression, it was only found unambiguously in roots, while levels in other tissues were nearly negligible (**Figure 6D**).

### Role of H^+^-PPases in Plant Tridemorph Tolerance

**FIGURE 7:**
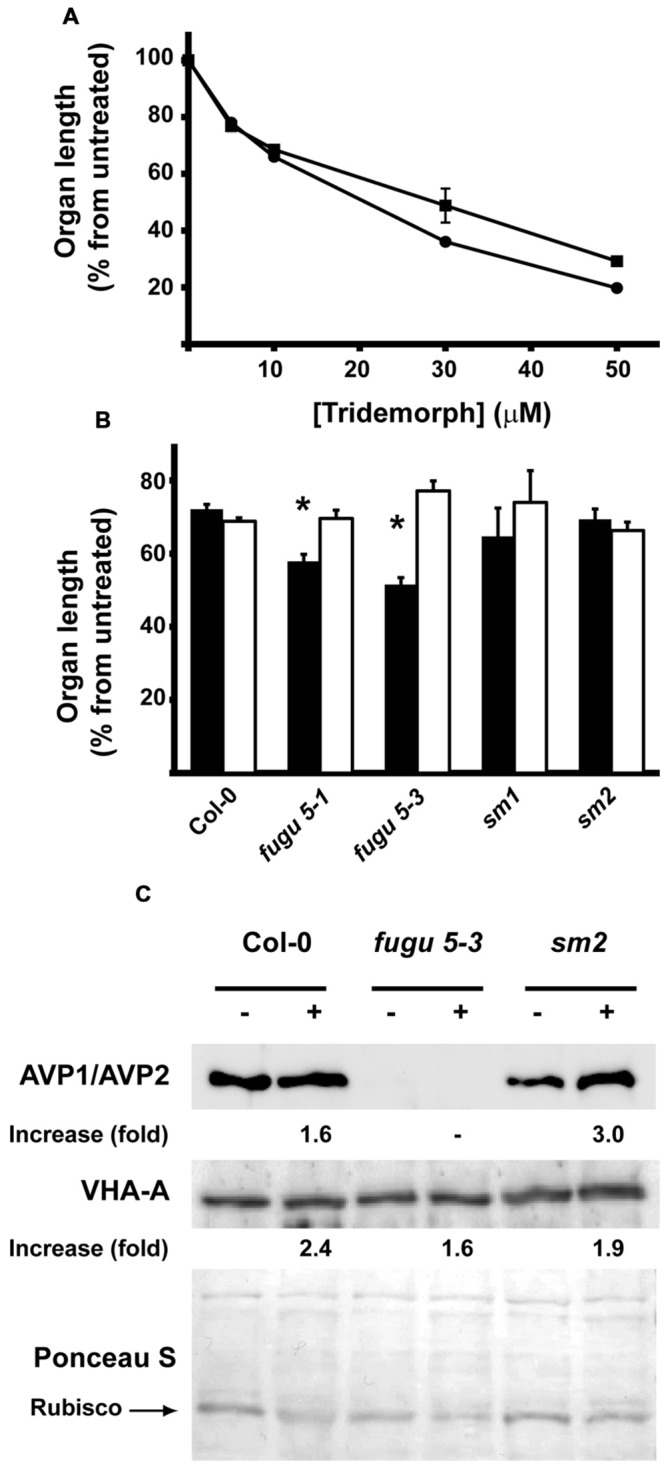
**Effect of tridemorph addition on seedling root and hypocotyl elongation. (A)** Length of roots and hypocotyls on wild-type *A. thaliana* as a function of fungicide concentration. Circles, root length; squares, hypocotyl length. **(B)** Length of roots and hypocotyls on wild-type and H^+^-PPase mutants of *A. thaliana.* Solid bars, roots; empty bars, hypocotyls. **(C)** Polypeptide levels of AVP1 and V-ATPase in tridemorph treated wild-type and pyrophosphatase mutants. Seeds were germinated and grown in the dark in phytagel-MS medium supplemented with 1% sucrose for 14 days prior analysis. Length measurements done by image analysis using NIH-ImageJ. Asterisks denote significant differences at *p* ≤ 0.05 using unpaired *t*-tests. Equal amounts of whole plant extracts were separated by SDS-PAGE, electroblotted onto nitrocellulose and decorated with antibodies recognizing the subunit A from V-ATPase (VHA-A) or AVP1. The protein band corresponding to RUBISCO large subunit (arrow) and the whole lanes stained with Ponceau S were used as loading controls with similar results. Those obtained with whole lanes are shown.

Once the expression of the three *AVP* isoforms was settled, we met the question of whether H^+^-PPases were involved in *A. thaliana* fungicide tolerance (**Figure 7**). We found that tridemorph produced a dose-dependent growth retardation effect in developing seedlings grown in the dark. This effect was noticeable in both hypocotyl and root lengths as reductions in both parameters nearing *ca* 40% at a concentration of 10 μM tridemorph (**Figure 7A**). To answer which of the H^+^-PPases was involved in fungicide tolerance, we treated wild-type (Columbia ecotype) and two *null* alleles of either *avp1* (*fugu 5-1* and *fugu 5-3*) or *avp2* (*sm1* and *sm2*) mutants with 10 μM tridemorph. Plants mutant for *AVP3* were cast-off from this study since expression levels of this gene, even in developing tissues, was nearly undetectable. As shown on **Figure 7B**, all seedlings displayed a remarkable reduction in both root and hypocotyl length upon tridemorph treatment. However, *fugu 5-1* and *5-3* mutants showed a significantly greater reduction in root length compared with either wild-type or *avp2* mutants, while these latter ones showed no differences between them (**Figure 7B**). In the case of hypocotyl length, none of the mutants showed any differences compared with a wild-type.

Next, we wanted to find out if H^+^ PPase or V-ATPase polypeptides levels varied as a response to tridemorph treatment. For this purpose, we measured the levels of polypeptides in whole seedling extracts by *Western* blot compared to levels of the small subunit of the ribulose 1,5 biphosphate carboxilase-oxygenase (RuBisCO; **Figure 7C**). Unfortunately, we were unable to perceive detectable levels of AVP2 even in *avp1 null* backgrounds, despite the use of two different polyclonal rabbit antibodies recognizing H^+^-PPases (**Figure 7C**, upper panel and data not shown). Effects on V-ATPases, as measured using the signal from the polypeptide corresponding to their catalytic subunit A as a marker of the holoenzyme, were apparent but modest in all mutants (**Figure 7C**, lower panel). Similarly, H^+^-PPase abundance increased discreetly in the case of wild-type plants treated with tridemorph; however, the accumulation of AVP1 augmented distinctly in the case of *avp2 null* mutants (**Figure 7C**, upper panel).

## Discussion

In this report, we have shown that tridemorph, an amine-type fungicide, can induce a cell death process in yeast that shows typical markers of apoptosis. In addition, tridemorph was able to induce cell cycle arrest to a certain extent. Apoptotic demise in fungi has been controversial for some time but it is now widely accepted, among other reasons, because ortholog genes of the basic machinery have been found and proved to be functional in yeast (Carmona-Gutierrez et al., 2010). Remarkably, death induction by tridemorph was independent of *YCA1*, the only metacaspase identified in the yeast genome. PCD in yeast has been shown to depend on *YCA1* in some cases (e.g., de Castro et al., 2011), but it is dispensable for others (Guaragnella et al., 2010; Hoeberichts et al., 2010), much in the same way as it happens in mammalian cells (Guaragnella et al., 2010; Hoeberichts et al., 2010). At any rate, little is known about the mechanisms of this type of cell death in yeast. Thus, tridemorph can be instrumental in the delineation of the mechanisms behind Yca1p-independent apoptosis in yeast.

For many years, it was considered that the main effect of the accumulation of 8-dehydrosterols, like any other abnormal sterols, was an increase in the permeability of the plasma membrane and that this latter effect was the actual cause for their death induction capability (Bard et al., 1978; Abe and Hiraki, 2009). There is no doubt that plasma membrane physical characteristics are affected upon extensive replacement of naturally occurring ergosterol by abnormal lipid species. However, little has been done along these years to determine if plasma membrane-associated effects are the actual cause of cell death and which mechanism it encompasses. Some years ago, it was shown that changes in physicochemical characteristics of the plasma membrane by abnormal sterols in *U. maydis* did not provide an explanation for the fungicidal action of these lipids (Hernandez et al., 1997). Following this, the present report showed that tridemorph-treated cells did not accumulate PI initially, which ruled out necrosis as the plausible mechanism of cell death under these conditions, but also questioned the long-held view of an increase in permeability at the plasma membrane level. Furthermore, the ability to reduce fungicide-induced cell death dramatically by expression of an alternative H^+^ pump that localizes preferentially in internal membranes, such as the *TcGFP-AVP1* construct (Drake et al., 2010), proved that at least part of the actual cellular mode of action of 8-dehydrosterols is located at the vacuole, though probably not exclusively. It must be mentioned here that, although *erg2Δ* cells showed many phenotypes in common with tridemorph treatment, the former did not show any detectable PCD. The reasons for this discrepancy are unknown, but may be related to either secondary mutations that help these cells to cope with the presence of abnormal sterols or because tridemorph has other targets independently of the Erg2p protein.

It has been proposed that 14α-methylated sterols exert part of their effects through inhibition of the vacuolar H^+^-ATPase in budding yeast and *C. albicans* (Zhang and Rao, 2010; Zhang et al., 2010). However, this kind of data are not necessarily applicable to all types of abnormal sterols; for example, the proton transport across plasma membrane vesicles was found to be uncoupled by 8-dehydrosterols but showed no differences by the presence of biologically active levels of 14α-methylated sterols in *U. maydis* (Hernandez et al., 1998). Here we showed that tridemorph, a fungicide inducing the accumulation of 8-dehydrosterols in yeast (Baloch and Mercer, 1987), causes the same effects on V-ATPase as a mutation in *ERG2* gene (Hernandez et al., 2015), i.e., a reduction in the proton transport capacity due to uncoupling from ATPase hydrolysis, but without the concomitant inhibition in ATP hydrolytic activity observed with 14α-methylated sterols. This uncoupling is also concomitant to protease sensitivity (Hernandez et al., 2015). Not surprisingly then, a mutation in *PEP4*, the gene encoding the master protease at the yeast vacuole, resulted in increased tolerance to tridemorph. Fungicide resistance is a growing concern in agriculture and, thus, information on possible mutational targets can be of great importance in order to understand field results on pest resistance.

In plant vacuoles, V-ATPases and H^+^-PPases share a common location. Recently, we have shown that the latter type of pumps is sufficient for acidifying internal compartments (Perez-Castiñeira et al., 2011). When grown on glucose, YPC3 yeast cells expressing *TcGFP-AVP1* reflects closely the situation found in a plant cell in terms of PPi homeostasis and proton transport across the tonoplast. We previously reported that this chimera is able to pump protons and, thus, functionally substitute vacuolar V-ATPase (Perez-Castiñeira et al., 2011); moreover, it could provide a means to test the influence of abnormal sterols in endomembrane traffic (Hernandez et al., 2015). We therefore hypothesized that this transporter may be an important factor in plant EBI fungicide tolerance. Indeed, the greater tolerance to an amine fungicide found in these metabolically engineered fungal cells concurs with what is usually observed in plants. Conversely, *A. thaliana* mutants lacking AVP1 polypeptides were more sensitive to tridemorph, specifically at the roots. This is in agreement with earlier reports showing that *A. thaliana hydra1* mutants (*null* mutants for the gene encoding sterol-Δ^8^, Δ^7^-isomerase, one of the two identified targets of tridemorph, the other being the sterol-Δ^14^-reductase) were affected in seedling root development (Souter et al., 2002). Also, it is consistent with *AVP1* gene being the most expressed gene isoform throughout the plant and especially in root tissues. This underlines the importance of the vacuole and, hence, their luminal acidification mechanisms, in root elongation (Barrada et al., 2015). On the other hand, *AVP2* is distinctly expressed in stems and, therefore, an influence on hypocotyl length on tridemorph treated *avp2* seedlings could be foreseen. However, since expression of *AVP2* is in the same order as that of *AVP1* in this organ, lack of any effect on hypocotyl elongation can be ascribed to compensation by AVP1 activity, in agreement with greater polypeptide amounts observed in *avp2* mutants upon tridemorph treatment. A contribution by AVP3 cannot be discarded; however, the levels of *AVP3* mRNA compared with those of *AVP1* where far too dissimilar to credit *AVP3* with any major influence. All in all, the presence of a double set of H^+^ pumps can be a factor that helps to explain why plants can tolerate this kind of compounds, even though they affect the sterol composition of their cell membranes (He et al., 2003).

## Author Contributions

AH contributed with the design of the study, experimental work, and manuscript writing; RH-P, JM, TA, GL-L, and JP-C contributed experimental work; AH, PN, FV and AS coordinated and financed the present study.

## Conflict of Interest Statement

The authors declare that the research was conducted in the absence of any commercial or financial relationships that could be construed as a potential conflict of interest.

## Abbreviations

A_600_: absorbance at 600 nm
GFP: green fluorescent protein
H^+^-PPase: H^+^- translocating pyrophosphatase
IP: immunoprecipitation
PI: propidium iodide
PPi: inorganic pyrophosphate
UPR: unfolded protein response
