# Supplementary figures and images for: Vacuolar H+-Pyrophosphatase AVP1 is Involved in Amine Fungicide Tolerance in Arabidopsis thaliana and Provides Tridemorph Resistance in Yeast
Source: Front Plant Sci. 2016 Feb 9;7:85. doi: 10.3389/fpls.2016.00085 (PMC4746327; doi:10.3389/fpls.2016.00085)

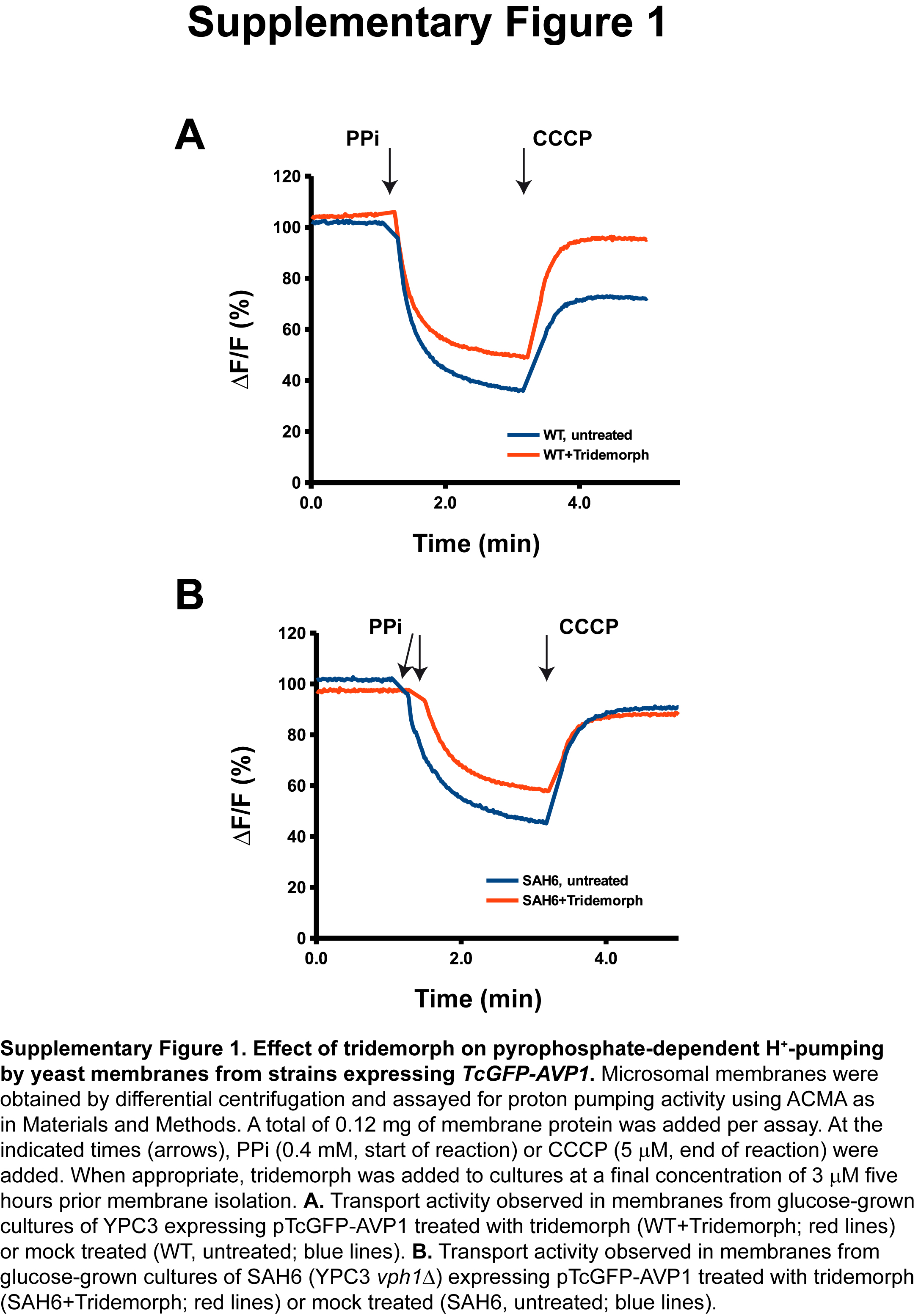

Supplement: Supplementary file 1 [file Image_1.JPEG]
